# Supplementary material for: A One Health assessment of antimicrobial-resistant Enterobacterales in migratory little stints (Calidris minuta) and aquatic ecosystems in the Kenyan Rift Valley
Source: PLoS One. 2026 Mar 27;21(3):e0336315. doi: 10.1371/journal.pone.0336315 (PMC13028371; doi:10.1371/journal.pone.0336315)
Supplement: Attachment — Submitted filename: Response to Reviewers.docx [file pone.0336315.s003.docx]

**View Letter**

| Date: | Nov 23 2025 08:49PM |
| --- | --- |
| To: | "CATHERINE WANGUI MBUTHIA" katieymbuthia@gmail.com |
| From: | "PLOS ONE" plosone@plos.org |
| Subject: | PLOS ONE Decision: Revision required [PONE-D-25-57615] |

PONE-D-25-57615

One Health assessment of antimicrobial-resistant Enterobacterales and ESKAPE pathogens in little stints (Calidris minuta) and aquatic ecosystems of the Kenyan Rift Valley

PLOS ONE

Dear Dr. MBUTHIA,

Thank you for submitting your manuscript to PLOS ONE. After careful consideration, we feel that it has merit but does not fully meet PLOS ONE’s publication criteria as it currently stands. Therefore, we invite you to submit a revised version of the manuscript that addresses the points raised during the review process.

Please submit your revised manuscript by Jan 07 2026 11:59PM. If you will need more time than this to complete your revisions, please reply to this message or contact the journal office at [plosone@plos.org](mailto:plosone@plos.org). When you're ready to submit your revision, log on to https://www.editorialmanager.com/pone/ and select the 'Submissions Needing Revision' folder to locate your manuscript file.

Please include the following items when submitting your revised manuscript:

- A rebuttal letter that responds to each point raised by the academic editor and reviewer(s). You should upload this letter as a separate file labeled 'Response to Reviewers'.
- A marked-up copy of your manuscript that highlights changes made to the original version. You should upload this as a separate file labeled 'Revised Manuscript with Track Changes'.
- An unmarked version of your revised paper without tracked changes. You should upload this as a separate file labeled 'Manuscript'.

If you would like to make changes to your financial disclosure, please include your updated statement in your cover letter. Guidelines for resubmitting your figure files are available below the reviewer comments at the end of this letter.

If applicable, we recommend that you deposit your laboratory protocols in protocols.io to enhance the reproducibility of your results. Protocols.io assigns your protocol its own identifier (DOI) so that it can be cited independently in the future. For instructions see: <https://journals.plos.org/plosone/s/submission-guidelines#loc-laboratory-protocols>. Additionally, PLOS ONE offers an option for publishing peer-reviewed Lab Protocol articles, which describe protocols hosted on protocols.io. Read more information on sharing protocols at <https://plos.org/protocols?utm_medium=editorial-email&utm_source=authorletters&utm_campaign=protocols>.

We look forward to receiving your revised manuscript.

Kind regards,

Mabel Kamweli Aworh, DVM, MPH, PhD. FCVSN
Academic Editor
PLOS ONE

Journal Requirements:

When submitting your revision, we need you to address these additional requirements.

1. Please ensure that your manuscript meets PLOS ONE's style requirements, including those for file naming. The PLOS ONE style templates can be found at

https://journals.plos.org/plosone/s/file?id=wjVg/PLOSOne_formatting_sample_main_body.pdf and <https://journals.plos.org/plosone/s/file?id=ba62/PLOSOne_formatting_sample_title_authors_affiliations.pdf>

2. We note that your Data Availability Statement is currently as follows: [All relevant data are within the manuscript and its Supporting Information files- I have added Supporting information-Compressed/Zip File Archive (S1 and S2 Tables that have all the sample descriptives and AMR profiles of all isolates respectively, for all the study areas, sample sources and cohorts.

Please confirm at this time whether or not your submission contains all raw data required to replicate the results of your study. Authors must share the “minimal data set” for their submission. PLOS defines the minimal data set to consist of the data required to replicate all study findings reported in the article, as well as related metadata and methods -(<https://journals.plos.org/plosone/s/data-availability#loc-minimal-data-set-definition)-Added>

For example, authors should submit the following data:

- The values behind the means, standard deviations and other measures reported;
- The values used to build graphs;
- The points extracted from images for analysis- Attached in the Supporting information-Compressed/Zip File (Tie-corrected Kruskal-Wallis Test Analysis)

Authors do not need to submit their entire data set if only a portion of the data was used in the reported study.

If your submission does not contain these data, please either upload them as Supporting Information files or deposit them to a stable, public repository and provide us with the relevant URLs, DOIs, or accession numbers. For a list of recommended repositories, please see <https://journals.plos.org/plosone/s/recommended-repositories-Attached> in the Supporting information

If there are ethical or legal restrictions on sharing a de-identified data set, please explain them in detail (e.g., data contain potentially sensitive information, data are owned by a third-party organization, etc.) and who has imposed them (e.g., an ethics committee). Please also provide contact information for a data access committee, ethics committee, or other institutional body to which data requests may be sent. If data are owned by a third party, please indicate how others may request data access.

3. We note that Figures 1A and 1B in your submission contain map images which may be copyrighted. All PLOS content is published under the Creative Commons Attribution License (CC BY 4.0), which means that the manuscript, images, and Supporting Information files will be freely available online, and any third party is permitted to access, download, copy, distribute, and use these materials in any way, even commercially, with proper attribution. For these reasons, we cannot publish previously copyrighted maps or satellite images created using proprietary data, such as Google software (Google Maps, Street View, and Earth). For more information, see our copyright guidelines: <http://journals.plos.org/plosone/s/licenses-and-copyright>.

We require you to either (1) present written permission from the copyright holder to publish these figures specifically under the CC BY 4.0 license, or (2) remove the figures from your submission:

1. You may seek permission from the original copyright holder of Figures 1A and 1B to publish the content specifically under the CC BY 4.0 license.

We recommend that you contact the original copyright holder with the Content Permission Form (http://journals.plos.org/plosone/s/file?id=7c09/content-permission-form.pdf) and the following text:

“I request permission for the open-access journal PLOS ONE to publish XXX under the Creative Commons Attribution License (CCAL) CC BY 4.0 (http://creativecommons.org/licenses/by/4.0/). Please be aware that this license allows unrestricted use and distribution, even commercially, by third parties. Please reply and provide explicit written permission to publish XXX under a CC BY license and complete the attached form.”

Please upload the completed Content Permission Form or other proof of granted permissions as an "Other" file with your submission.

In the figure caption of the copyrighted figure, please include the following text: “Reprinted from [ref] under a CC BY license, with permission from [name of publisher], original copyright [original copyright year].”

2. If you are unable to obtain permission from the original copyright holder to publish these figures under the CC BY 4.0 license or if the copyright holder’s requirements are incompatible with the CC BY 4.0 license, please either i) remove the figure or ii) supply a replacement figure that complies with the CC BY 4.0 license. Please check copyright information on all replacement figures and update the figure caption with source information. If applicable, please specify in the figure caption text when a figure is similar but not identical to the original image and is therefore for illustrative purposes only

The following resources for replacing copyrighted map figures may be helpful:
USGS National Map Viewer (public domain): http://viewer.nationalmap.gov/viewer/
The Gateway to Astronaut Photography of Earth (public domain): http://eol.jsc.nasa.gov/sseop/clickmap/
Maps at the CIA (public domain): https://www.cia.gov/library/publications/the-world-factbook/index.html and <https://www.cia.gov/library/publications/cia-maps-publications/index.html>
NASA Earth Observatory (public domain): http://earthobservatory.nasa.gov/
Landsat: http://landsat.visibleearth.nasa.gov/
USGS EROS (Earth Resources Observatory and Science (EROS) Center) (public domain): http://eros.usgs.gov/#
Natural Earth (public domain): <http://www.naturalearthdata.com/>

The maps (Figure 1 and Figure 2) were generated using QGIS software version 3.4, which is an open-source Geographic Information System licensed under the GNU General Public License (GPL). As per the QGIS Foundation, the software creators do not hold copyright over works produced by users. Therefore, as the author of the maps, I grant full permission for them to be published under the CC BY 4.0 license.

I have updated the figure captions within the manuscript to explicitly state the open-access data sources used (OpenStreetMap and HDX), ensuring that no copyrighted proprietary base maps (such as Google Maps or Esri) were utilized.

Updated Captions: ‘Maps were created by the authors using QGIS version 3.4. Administrative boundaries are sourced from the Humanitarian Data Exchange (licensed under CC BY 4.0). Lake boundaries and river networks are sourced from OpenStreetMap (available under the Open Database License). All other data points were collected by the authors. This figure is original and licensed under CC BY 4.0.’

If the reviewer comments include a recommendation to cite specific previously published works, please review and evaluate these publications to determine whether they are relevant and should be cited. There is no requirement to cite these works unless the editor has indicated otherwise. 

Please review your reference list to ensure that it is complete and correct. If you have cited papers that have been retracted, please include the rationale for doing so in the manuscript text, or remove these references and replace them with relevant current references. Any changes to the reference list should be mentioned in the rebuttal letter that accompanies your revised manuscript. If you need to cite a retracted article, indicate the article’s retracted status in the References list and also include a citation and full reference for the retraction notice-I have not cited any retracted paper

Additional Editor Comments:

In addition to addressing the reviewers comments, please highlight key limitations of the study as the last paragraph of the Discussion section. Also ensure that 80% of the reference list is within the last five years. – Included in Lines 311-318


[Note: HTML markup is below. Please do not edit.]

**REVIEWERS' COMMENTS:**

Reviewer's Responses to Questions

**Comments to the Author**

1. Is the manuscript technically sound, and do the data support the conclusions?

The manuscript must describe a technically sound piece of scientific research with data that supports the conclusions. Experiments must have been conducted rigorously, with appropriate controls, replication, and sample sizes. The conclusions must be drawn appropriately based on the data presented.

Reviewer #1: Yes

Reviewer #2: Yes

2. Has the statistical analysis been performed appropriately and rigorously?

Reviewer #1: Yes

Reviewer #2: I Don't Know

3. Have the authors made all data underlying the findings in their manuscript fully available?

The [PLOS Data policy](http://www.plosone.org/static/policies.action#sharing) requires authors to make all data underlying the findings described in their manuscript fully available without restriction, with rare exception (please refer to the Data Availability Statement in the manuscript PDF file). The data should be provided as part of the manuscript or its supporting information, or deposited to a public repository. For example, in addition to summary statistics, the data points behind means, medians and variance measures should be available. If there are restrictions on publicly sharing data—e.g. participant privacy or use of data from a third party—those must be specified.

Reviewer #1: Yes

Reviewer #2: Yes

4. Is the manuscript presented in an intelligible fashion and written in standard English?

PLOS ONE does not copyedit accepted manuscripts, so the language in submitted articles must be clear, correct, and unambiguous. Any typographical or grammatical errors should be corrected at revision, so please note any specific errors here.

Reviewer #1: Yes

Reviewer #2: Yes

5. Review Comments to the Author

Please use the space provided to explain your answers to the questions above. You may also include additional comments for the author, including concerns about dual publication, research ethics, or publication ethics. (Please upload your review as an attachment if it exceeds 20,000 characters)

Reviewer #1: General Comment

This paper offers truly important insights into how migratory birds play a role in spreading antimicrobial resistance. Its findings are vital for guiding public health policies and environmental strategies against AMR. What's particularly exciting is that this study uncovers a link between the little stint bird and AMR, thoroughly addressing a big gap in what we currently know. Looking ahead, authors should consider sequencing their isolates, as a future study. It would provide much deeper genomic insights, especially into the mobile genetic elements that carry or share these resistance genes.

Specific Comments

Line 242–243: The table layout appears cluttered, making it hard to read, especially at publication scale. Also, consider defining these abbreviations (for example “C1”, “C2”) mentioned in the caption. Did an overhaul of Table 1 and C1 and C2 -cohorts 1 and 2 are no longer included- Lines 244-247

Line 256: Insert a space between “1” and 2. Rewritten the paragraph-Lines 250-257

Lines 261–262: Similar to Table 1, define all abbreviations for clarity. Improve table formatting for readability-Done lines 260-263 under key

Lines 274–278: Good you mentioned, potential AMR factors such as human interactions at hot springs, rivers shared with livestock, and salt pans effluent. Consider elaborating on how these factors directly contribute to AMR in migratory birds in the regions. Expand the discussion to explain the mechanisms or pathways through which these factors may directly or indirectly influence AMR transmission or persistence-Rephrased at lines 281-287
Lines 442–443: Review reference formatting (for instance citations, 35 and 39) and ensure consistent use of the appropriate citation style as required by the journal throughout the reference list-Removed Ref 39 but fixed Ref 35

Reviewer #2: The topic of the study is definitely of interest to the AMR community. Minor corrections are suggested as enumerated below

1. Paragraph starting at line 64. Introducing a statement or 2 describing how the little stint (Calidris minuta) specifically feeds and generally interacts with the niche upon arrival at lakes Magadi and Bogoria will be informative- Corrected at lines 67-71.
2. Line 77. "While anthropogenic activities are recognized as a key driver of AMR dissemination by migratory birds globally,", kindly provide reference for this- Done-Line 83
3. Line 94. "Anthropogenic activity is substantial due to the commercial trona and soda ash mining industry." Knowing that PLoS One is a generalist publication, the authors might wish to clarify to readers how the commercial trona might contribute to AMR risk at this site-This is explicitly explained in the discussion (Line 270-276), and Lines 281-287 rather than in the study area description
4. Line 99. "Human interactions with the lake are minimal, with the agro-pastoralists Tugens and animals (domestic and wildlife) accessing the freshwater springs by the lakes." At present, I am not convinced, as per this statement, that the human interaction due to the agro-pastoralists and their animals are minimal (no evidence provided) or minimal compared to lake Magadi. The authors should provide clarifying evidence. Paraphrased at line 107-110. Since the main bodies of both lakes are unsuitable for hydration or domestic use due to their extreme chemistry, significant interaction between humans, livestock, and *C. minuta* occurs at the surrounding peripheral freshwater sources, including springs, rivers, marshes, and boreholes.
5. Line 117. Reporting the results of number of samples collected under the methods section is inappropriate. Such data should be in the result section. Corrected, now at Lines 211-214

6. Line 120. We are told that water samples were drawn from lakes, hot springs, rivers, and springs [4 sources] used by the little stint. What we are not told is whether the 48 samples that was eventually collected was achieved using random or other form of sampling from these 4 sources. Please clarify. Corrected-Line 137- Water samples were collected using criterion purposive sampling
7. At line 153, "They included; ampicillin (AMP, 10 μg), amoxicillin-clavulanic acid (AMC, 30 (20:10) μg), cefepime (FEP, 30 μg), cefotaxime (CTX, 30 μg), ceftriaxone (CRO, 30 μg), cefuroxime (CXM, 30 μg), meropenem (MEM, 10 μg), ciprofloxacin (CIP, 5 μg), tetracycline (TE, 30 μg), chloramphenicol (C, 30 μg), gentamicin (CN, 10 μg), trimethoprim sulfamethoxazole (SXT, 25 (1.25: 23.75). The authors, might explain to PONE readers what the numbers with the μg units mean and why μg is missing for trimethoprim sulfamethoxazole-Corrected at lines 172 and 177 A panel of 12 antimicrobial disks (Oxoid, Basingstoke, UK), representing 8 drug classes of varying concentrations (μg)….. sulfamethoxazole-trimethoprim (SXT, 25 μg).
8. Full legend information should accompany each table. For example, the authors should not leave readers to assume exactly what C1/C2 means. In Table 1, the value of the data within and outside brackets in each cell should be defined at the top of the column-Edited Table 1 (cohort is no longer part of this table) and written full cohort in Table 2

9. The authors should introduce a section on limitations encountered in this study. Limitations include lack of molecular studies to achieve source attribution for the bacteria isolated as well as determine dissemination pathways. Though the authors have tried to indirectly and subtly link the Rift Valley niche to AMR pathogens in these birds by mentioning environmental stewardship. Evidence for such is not presented in this study- Lines 316-323

6. PLOS authors have the option to publish the peer review history of their article ([what does this mean?](https://journals.plos.org/plosone/s/editorial-and-peer-review-process#loc-peer-review-history)). If published, this will include your full peer review and any attached files.

If you choose “no”, your identity will remain anonymous but your review may still be made public.

**Do you want your identity to be public for this peer review?** For information about this choice, including consent withdrawal, please see our [Privacy Policy](https://www.plos.org/privacy-policy).

Reviewer #1: No

Reviewer #2: No

[NOTE: If reviewer comments were submitted as an attachment file, they will be attached to this email and accessible via the submission site. Please log into your account, locate the manuscript record, and check for the action link "View Attachments". If this link does not appear, there are no attachment files.]

To ensure your figures meet our technical requirements, please review our figure guidelines: https://journals.plos.org/plosone/s/figures

You may also use PLOS’s free figure tool, NAAS, to help you prepare publication quality figures: https://journals.plos.org/plosone/s/figures#loc-tools-for-figure-preparation.

NAAS will assess whether your figures meet our technical requirements by comparing each figure against our figure specifications.
